# A Perspective on Software Intelligence for Autonomous Transformations in Biomedical Data and Knowledge

**DOI:** 10.1002/lrh2.70063

**Published:** 2026-01-19

**Authors:** Vivek Navale

**Affiliations:** ^1^ Center for Information Technology, National Institutes of Health Bethesda Maryland USA

**Keywords:** intelligent biomedical platform, intelligent data agents, multi‐agent system, software intelligence, software learning

## Abstract

**Introduction:**

Persistent knowledge is essential for propagating the learning health system (LHS) cycle. Integral to the cycle are iterative transformations of data into knowledge. However, human efforts to undertake these transformations are increasingly challenged when dealing with larger data scales and complexities. Data sets within repositories and archives are often underutilized unless specifically requested for research programs. Specialized software algorithms (agents) can use existing knowledge for learning tasks, explore their environment, discover and create goals, and interact with humans.

**Methods:**

This paper examines the potential role of software intelligence for autonomous transformations of data and knowledge. Agents can perform various goal‐directed tasks. Multi‐agent systems can be utilized for data collection, description, preparation, modeling, and knowledge‐mining tasks. Knowledge representation, ontologies, semantic web standards, knowledge bases, and graphs can lead to a higher level of directed learning. Agents can develop reasoning abilities and self‐generate goals by leveraging semantic relationships between various datasets.

**Results:**

A conceptual framework for an intelligent biomedical platform (IBP) is proposed. The IBP comprises four layers: infrastructure (IS), user interface (UI), coordination system (CS), and data and knowledge (DK). It also integrates a network of multi‐agent systems for clinical decision‐making and knowledge‐mining tasks. Intelligence in the platform results from the interaction of the IS, UI, CS, and DK agents. These agents can implement multiple inferential steps using the data and knowledge within accessible repositories. Large language models can be integrated with various knowledge resources and domain‐specific databases, thereby improving the accuracy of results.

**Conclusion:**

An IBP supported by a multi‐agent system can enhance the autonomous transformation of data and knowledge. Including software intelligence within current repositories and archives enhances data reuse and the generation of new knowledge. With the addition of software reasoning capabilities in biomedical platforms, the LHS cycle can be efficiently propagated to aid in newer biomedical discoveries.

## 1 Introduction

Knowledge is a keystone for the learning health system (LHS) cycle. It involves iterative transformations from data to knowledge that can continuously improve performance [1]. Human efforts to undertake these transformations increasingly face challenges when dealing with complex data at larger scales. Data sets within repositories and archives can remain underutilized unless they are specifically required for a particular research program.

Our current understanding of the human mind is based on knowledge of evolutionary biology, anatomy, physiology, adaptive brain function, and the bodys response to sensory stimuli. Exactly how the human mind processes information is an area of considerable research interest, and a significant challenge is to develop computational models that approximate the human mind. One of the theories presented in cognitive neuroscience is the Global Workspace Theory (GWT). It states that several independent, special‐purpose processes in the human brain coalesce within a ‘Global Workspace’ to achieve human cognition [2].

The biologically inspired Learning Intelligent Distribution Agent (LIDA) model implements the GWT theory. Within the LIDA model, tasks are carried out by small pieces of software code known as “codelets,” which are analogous to processors in the GWT. Each codelet carries out tasks independently and serves as an autonomous agent within a computing environment. An application of the above model led to the development of Medical Agent X (MAX), which replicated cognitive functions relevant to medical diagnosis. MAX focused on listing possible causes for patients' conditions, followed by reasons for ruling out certain identified possibilities [3].

Recent advances in deep learning models have enabled the extraction of meaningful information from data at various levels of abstraction, resulting in end‐to‐end learning [4]. These models, referred to as foundation models (FM), are pre‐trained with large unlabeled datasets using a self‐supervised learning technique with no human involvement. Large language models (LLMs) are a type of “FM” models that can learn using natural language processing (NLP) methods and generate responses to text‐based prompt queries [5].

Examples of FM include the Bidirectional Encoder Representation Transformer (BERT) and the Generative Pre‐trained Transformer (GPT) series, with applications in clinical NLP, medical image analysis, text‐to‐image generation, and medical computer vision [6].

LLMs can be programmed as part of a single‐agent system for tackling a broad range of tasks. Complex tasks benefit from deploying LLM‐based multiple agents. Powered by perception, interaction, memory, and reasoning modules present in individual agents, a multi‐agent system (MAS) can contribute to software development, industrial engineering, gaming, and automating scientific experiments [7].

Recent work has led to the development of an AI co‐scientist, a MAS that can assist scientists in uncovering new knowledge, generating novel hypotheses, and planning experiments for studying drug repurposing, novel target discovery, and explaining mechanisms of bacterial evolution and antimicrobial resistance [8].

### 1.1 Problem of Interest

Underutilization of biomedical data in repositories and the bottleneck resulting from human‐dependent data to knowledge transformations impede the acceleration of the LHS cycle. This paper is a perspective on how to enable autonomous, intelligent transformation of biomedical data and knowledge within existing repositories and platforms, thereby enhancing data reuse and accelerating knowledge creation without requiring constant human intervention.

In light of recent developments in active learning (AL) software, MASs, and LLMs, this paper examines the potential role of software intelligence in developing MAS for autonomous data and knowledge transformation. It also provides a conceptual framework for an intelligent biomedical platform (IBP) applicable to clinical decision‐making and knowledge mining.

Specifically, this work attempts to address broader questions: Can software algorithms possess enhanced reasoning capabilities analogous to those of the human mind? Can computational intelligence facilitate the autonomous transformation of data and knowledge in repositories, thereby strengthening the LHS cycle more efficiently?

## 2 Method

A review of AL in software, the functional role of software intelligence in agent‐based systems, AI‐based autonomous systems, and knowledge mining applications leads to the conceptualization of an IBP.

### 2.1 AL Software

Traditionally, software algorithms are developed for specified tasks. However, AL software can explore its environment, discover and create its goals, and interact with humans [9]. Learning can include goal selection and execution. AL has been utilized in robotics with the adoption of a Self‐Adaptive Goal Generation—Robust Intelligent Adaptive Curiosity (SAGG‐RIAC) architecture [10].

**FIGURE 1:**
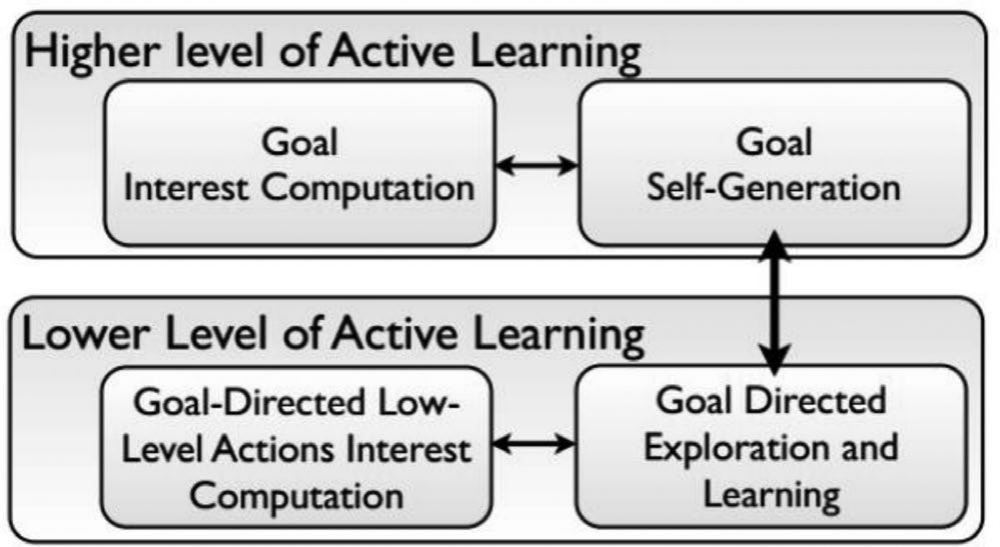
Illustrates a learning concept based on the SAGG‐RIAC architecture [10].

Figure 1 illustrates the SAGG‐RIAC architecture, where robots actively sample parametrized policies to learn goal exploration efficiently and progressively explore tasks of increasing learning complexity.

Using the above concept, specialized algorithms called “Agents” can perform a wide range of goal‐directed actions with varying degrees of autonomy and collaboration in biomedical research. For example, several agents can be involved in brainstorming a solution to a problem posed by human researchers; agents can provide expert consultation by interacting with a human scientist, utilizing domain knowledge and tools; two teams of agents can be engaged in research debates; and other agents can participate in round table discussions for decision‐making activities [11].

Agents can also learn knowledge representation, ontologies, semantic web standards, knowledge bases, and graphs [12]. The knowledge gained by learning semantic relationships between various datasets can enable proactive decision‐making in healthcare [13]. For example, knowledge graphs for clinical diagnosis have been developed by using a hierarchical MAS enabled by semantic‐driven entity extraction and relationship reconstruction from unstructured medical texts. A framework integrating BioBERT with medical ontologies (SNOMED‐CT, UMLS), LLMs, and MAS demonstrated automated discovery of semantic relationships across 362 diseases [14]. Similarly, in radiology visual question answering, MAS with contextual information, multimodal reasoning, and answer validation capabilities achieved significant improvements in resolving diagnostic ambiguities [15].

Developing proactive healthcare systems will require the utilization of various relationships in different contexts within big data [13]. The National Institute of Health's National Center for Advancing Translational Sciences (NCATS) Biomedical Translator demonstrates an approach where autonomous relay agents perform semantic reasoning across more than 40 integrated knowledge resources to identify novel therapeutic hypotheses and disease‐drug relationships [16].

### 2.2 Functional Aspects of Software Intelligence

To function in a collaborative MAS environment, individual agents carry out defined tasks and communicate their results with other agents to achieve the higher‐level goals set forth by users. Such a system can be rule‐based to enable planning, resource management, decision support, data management, remote care, and self‐care activities [17]. Rule‐based MAS within a data platform has enabled physicians and patients to collaborate on the care and management of cancer [18].

In addition to the rule‐based approach, LLM‐supported agents can be used to autonomously deploy specialized tools for retrieving, processing, and synthesizing new information from clinical data, including text, radiology, histopathology, imaging, and genomic data [19].

The LLM‐based MAS collaboration has resulted in the development of Med Aide, a framework that enables intent‐aware information fusion and coordinated reasoning across specialized healthcare domains. Four different benchmarks across 17 types of medical intents, including pre‐diagnosis, diagnosis, medicament, and post‐diagnosis, were conducted to demonstrate that Med Aide outperformed current LLMs in medical proficiency and strategic reasoning capabilities [20].

### 2.3 AI‐Based Autonomous Systems

System‐level design and architecture for self‐improvement have been explored through the application of an AutoAI algorithm [21]. Raw data from Open‐Source Intelligence (OSINT) sources is ingested in AutoAI, followed by automated feature engineering training tasks for learning purposes that categorize and analyze the data.

An AI‐based autonomous data system will involve the fusion of AI and database technologies. NeurDB is an example of developing in‐database AI‐powered analytics, resulting in a fully autonomous data system that can dynamically adapt itself with little or even without human intervention [22]. Autonomous data association methods have also been used for intelligent information discovery and knowledge generation [23], and practical use cases of autonomous AI systems have been developed to analyze images of the retina for diagnosing diabetic eye disease (DED) at primary care clinics in the United States [24].

#### 2.3.1 Knowledge Mining

BERT‐based and NLP‐based constituent extraction techniques have been utilized for autonomous knowledge mining [25]. A knowledge graph‐based platform that combines searching and reasoning on biomedical knowledge resources with question‐and‐answer capabilities can effectively result in knowledge mining. The NCATS Biomedical Translator Program has implemented a knowledge‐based platform for exploring the relationship between an ensemble of knowledge resources [16].

The Translator architecture comprises an autonomous relay system, relay agents, knowledge providers (KPs), translator reasoner application programming interface (API), along with standards and reference implementation services [16].

Over 40 highly curated databases and ontologies (e.g., the Comparative Toxicogenomics Database and the Monarch Disease Ontology) serve as knowledge resources. APIs exist for the Translator to KPs including Big Cell Line Association Miner, BioThings Explorer, Columbia Open Health Data, Data‐Driven Ontology Toolkit plus diseases ontologies, Fanconi anemia, Global Ingredient Archival Systems, Integrated Clinical and Environmental Exposure Service, Gene Ontology, Monarch disease Ontology, Network Data Exchange, Probabilistic Graphical Models, Socio‐Environmental Exposure Services, and the Semantic MEDLINE Database [26]. An open‐source universal data model, named the Biolink Model, provides semantic harmonization and reasoning across the KPs [27].

The Biomedical Translator has been applied to study immune‐mediated inflammatory diseases, finding a relationship between Crohn's and Parkinson's disease, and to search for potential therapeutic drug candidates for treating drug‐induced liver injury [16].

## 3 Results

**FIGURE 2:**
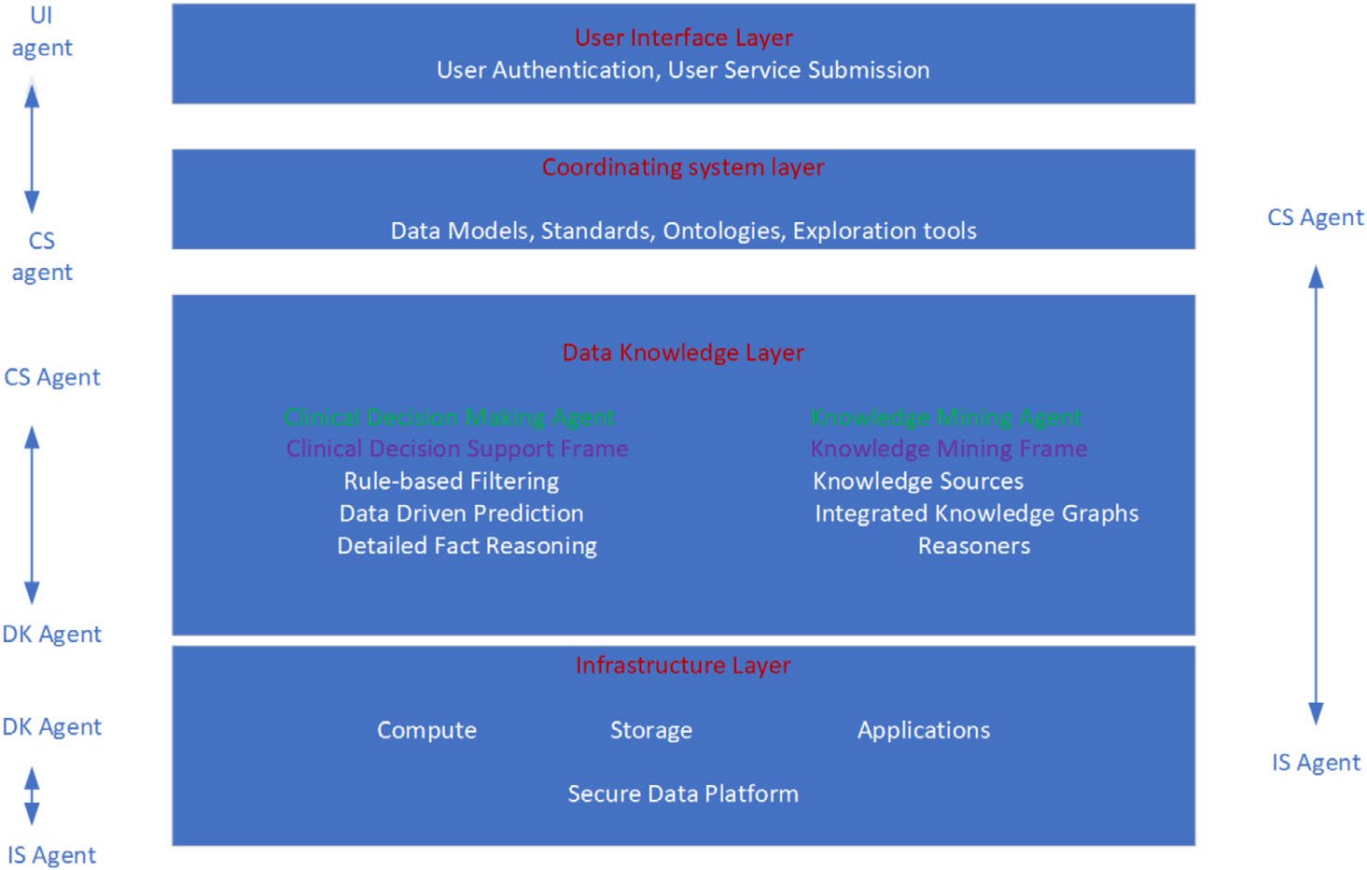
A conceptual framework for an intelligent biomedical platform representing a multi‐agent system that can be used for clinical decision support and knowledge mining. Abbreviations for various agents: User Interface (UI), Coordinating System (CS), Data Knowledge (DK), Infrastructure (IS), Clinical Decision Making (CDM), Knowledge Mining (KM) agents.

Based on the above review, a conceptual framework for an IBP is presented (Figure 2). The IBP comprises multiple layers. The four layers of the IBP are (a) the infrastructure (I) layer, (b) the coordination system (CS) layer, (c) the data and knowledge (DK) layer, and (d) the user interface (UI) layer. Each layer has a primary function in the platform, and the CS layer has a prominent role in establishing communication between the layers. Additionally, the layers are supported by intelligent agents (IAs), which are primarily responsible for achieving their specialized objectives.

### 3.1 IBP

Similar to currently existing biomedical platforms, the IBP relies on a scalable infrastructure and secure data services to ingest, process, validate, curate, store, and share data. Cloud computing supports the above requirements by providing rapid scalability and access to data resources [28]. Additional capabilities that comprise biomedical platforms include workflow(s), data analysis, visualization tools, and access to and from storage repositories [29]. The Cloud infrastructure is supported by machine learning (ML) algorithms for various applications [30], with services to process Big Data workloads [31].

However, with the currently available data platforms, mobilizing data for processing, storage, and management tasks requires significant human engagement. Developing software intelligence within the platform to autonomously carry out data life cycle tasks can enhance efficiency and accelerate knowledge creation within the learning health system cycle. The IBP is envisioned to comprise MAS with IAs that collaborate for clinical decision‐making and knowledge mining tasks.

The I‐layer is the foundation layer for the IBP, supported by cloud computing, storage repositories, applications, and services. The UI layer contains policies and procedures to determine the extent to which individual users and IAs will be granted access to the platform. Depending on the user (e.g., physician, researcher, patient), the UI layer facilitates search queries. Additionally, the UI layer is supported by a UI agent that interprets the user requests (e.g., simple queries, complex questions, data access requests) and communicates with the CS layer agent.

The CS layer comprises standards, ontologies, data‐driven models, and exploration tools. The CS agent supports the CS layer by receiving requests from the UI agent. With the help of the CS agent, the CS layer responds to service needs. It also communicates with multiple specialized agents with expertise in standards, ontologies, models, and data tools, responds to service needs, and coordinates with the DK agent to provide context recognition services.

The DK layer has two frames supported by a DK agent: Clinical Decision‐Making (CDM) and Knowledge Mining (KM). The DK agent communicates with the CS agent and then determines whether to activate the CDM and/or the KM frame. To develop the CDM frame, a three‐stage clinical decision support system framework can be applied [32]. The three stages involve rule‐based filtering and decision support (Stage 1), data‐driven predictive modeling (Stage 2), and detailed fact‐reasoning stages (Stage 3). Stage 1 references existing policies and recommendations from human experts. It can be implemented with rules, decision trees, and semantic structures. This stage is followed by Stage 2, which utilizes data‐driven predictive modeling and ML algorithms, and Stage 3, which focuses on the explainability supported by using heuristics, explainable AI, and explainable metamodeling techniques. A CDM agent supports the above activities and serves as the primary broker to the CS agent's needs by ascertaining the appropriate implementation process for individual service requests.

The IS layer contains data repositories and provides a common area where patient data is stored and access to agents is provided based on service needs and permission rights. The CDM agent coordinates the activities of other agents supporting the CDM frame, responding to “Events” and triggering the activation of a specific agent for the work. The feasibility of a coordinating agent that controls the activation of knowledge resources in a distributed reasoning environment was demonstrated [18].

The KM frame utilizes the NCATS Translator design discussed earlier. It comprises semantically annotated knowledge sources, Integrated Knowledge Graphs, and “Reasoners.” The KM frame is supported by a KM agent with an autonomous relay system that broadcasts queries and receives answers from multiple autonomous relay agents [16].

In the Translator design, a user query is expressed in natural language, which is then translated into a query graph in JSON format. The query graph can be delivered to relay agents for distribution to KPs that can respond to the query. The responses returned from the KPs are processed through reasoning, analytical, and inferential algorithms before being delivered to the user.

Intelligence in the IBP results from the interaction of UI, CS, DK, and IS agents. The agents carry out multiple inferential steps using data and knowledge from accessible sources, including domain‐specific repositories. Generative AI and LLMs can be coupled with the IBP to provide support for decision‐making, question answering, knowledge retrieval, and report generation [33].

## 4 Discussion

IBPs can use a combination of agents with specific functional capabilities. Interpreting NLP queries can be a focus area of expertise for UI agents. The CS agent can provide context recognition for transforming NLP into knowledge graph‐oriented queries. The CS agent can collaborate with the DK agent to handle text and images, which can be combined with additional capabilities that result in dialogue‐based knowledge [34].

The DK agent can activate the CDM agent for model training and provide support for visualization and predictive recommendations. The feasibility of using a model‐based training method has been demonstrated for hypertension, diabetes mellitus, and chronic heart failure [32].

The DK layer can also support a deep variational sequential Generative Biomarker Identification Learning (GERBIL) framework, which can automatically identify disease‐specific biomarker subsets. The application of the GERBIL framework was demonstrated for datasets from Gastric Cancer (GC), Epstein–Barr virus‐associated GC, and Intestinal Metaplasia [35].

Compared to traditional AI systems, multimodal LLMs integrate diverse data types to generate new content via Generative Artificial Intelligence (GenAI). A fully autonomous agent that integrates LLMs with dynamic tool execution and adaptive reasoning was developed for spatial biology research [36].

The use of GenAI in clinical work in gastroenterology was effective for tasks such as documentation, medical billing, scheduling, summarizing medical literature, and patient education. Complex tasks involving clinical reasoning for diagnostic and treatment decisions require careful human oversight when implementing recommendations from GenAI [37].

Current application constraints of commercial Gen AI for the clinical and biomedical sciences include a lack of an open‐source platform and interpretability questions related to how the results from GenAI were achieved for specific tasks. To address some of the above challenges, Biochatter, an open‐source Python framework, was developed for biomedical applications [38]. BioChatter integrates with BioCypher [39] and other databases that provide domain knowledge to perform retrieval‐augmented generation. Knowledge graph query generation demonstrated that the Biochatter prompt engine achieved higher accuracy than native LLMs.

### 4.1 MAS Design Considerations

Developing trustworthy MAS requires attention to areas involving bias reduction, formal and runtime validation and verification, transparency, and the explainability of actions, with a human‐in‐the‐loop interface as part of the design.

The Multi‐Agent Reinforcement Learning (MARL) method can be used for carrying out task actions, negotiations, and allocating resources [40]. Using MARL, agents learn collaborative strategies for environments similar to the proposed IBP. However, over‐ and underestimation of errors can result from MARL. Bias resulting from MARL can be corrected by using the Median Weighted Multi‐Agent Deep Deterministic Policy Gradient method [41].

Bias can also result when LLM‐based agents replicate or amplify cultural or demographic inequities [42]. Methods to alleviate social bias that have been studied include data augmentation, parameter tuning, and reinforced learning with human feedback [43]. Transparency and explainability can be enhanced by incorporating Chain‐of‐Thought (CoT) reasoning methods [44].

A Multi‐Objective within a Multi‐Agent framework (MOMA) that mitigates social bias in LLMs without significant performance compromise has been developed. Within the MOMA framework, multiple agents (masking, balancing, and task agents) are deployed to perform causal interventions on bias‐related content in the input questions [45].

To ensure safety and compliance, formal verification of the MAS design is important and can be conducted using an open‐source Model Checker for Multi‐Agent System (MCMAS). The MCMAS toolkit verifies MAS through a range of specified agent‐based logic [46]. The formal MAS verification methods can be further extended by applying the Rational Verification method. This method considers system component preferences and temporal logic properties to determine whether the MAS will act rationally by exhibiting individual agent properties within the system [47].

MARL decision‐making process is an important design consideration for a transparent MAS. Seven key metrics for assessing the explainability of the reinforcement learning method have been identified [48]. The metrics include Fidelity, Performance, Comprehensibility, Preferability, Actionability, Cognitive load, and Visualization. The authors also provide information on the general questions answered by the metric, with corresponding examples.

Other methods for enabling understanding of the agent's actions in the MAS can result from developing various Policy Graphs [49], which can be transformed into natural language that is interpretable by humans. The Assured Reinforced Learning Model Interrogation toolkit can be a useful resource [50] for identifying potential vulnerabilities within a deep reinforcement learning model.

Overall, the MARL method has the potential to enable agents to autonomously learn and perform tasks efficiently. The learning process is enhanced when human feedback is available to the agents, as part of Human‐in‐the‐Loop (HITL) within the MAS. Four phases of human involvement during agent development, learning, evaluation, and deployment have been identified [51]. Design robustness and explainability are enhanced when human experts contribute to the entire life cycle of MAS.

When designing LLM‐based MAS, safety and security measures are needed. Malicious prompt engineering can result in outputs that are misleading and erroneous. To mitigate the risk, role‐based filters should be implemented that can detect and filter malicious content in the input and output of each agent within a MAS [52].

## 5 Conclusion

Software intelligence can be developed at lower and higher levels. Lower levels can involve individual agents carrying out specific tasks to collect, manage, store, and access data. Higher‐level agents can focus on the contextual understanding of data, which can lead to counterfactual experimentation.

An IBP supported by MAS can enhance the autonomous transformation of data and knowledge. A trustworthy MAS requires tools to support bias reduction, provide methods for validation, verification, transparency, and explainability, along with design considerations that include HITL for oversight and safeguards.

Specific applications of MAS can be developed with reasoning capabilities to support clinical decision‐making and knowledge mining. Agents can use rule‐based filtering, data‐driven modeling, and detailed fact reasoning. Agents specializing in knowledge graph techniques can combine searching and reasoning of biomedical resources, leveraging LLMs and Generative AI for knowledge mining purposes.

Integrating reasoning capabilities within biomedical platforms can enable the learning health system cycle to propagate efficiently, promoting newer discoveries in biomedical science. Soon, autonomous scientific discovery can become a reality.

## Disclosure

The findings and conclusions presented in this paper are those of the author and do not necessarily reflect the views of the National Institutes of Health or the U.S. Department of Health and Human Services.

## Conflicts of Interest

The author declares no conflicts of interest.

## Data Availability

No data are required for this work.
